# Foreign Quackeries and the Drug-Inspection Law

**Published:** 1852-10

**Authors:** 


### THE MEDICAL EXAMINER.

PHILADELPHIA, OCTOBER, 1852.

### FOREIGN QUACKERIES AND THE DRUG-INSPECTION LAW.

A recent case has raised the question of the admission of foreign quack medicines under the drug-inspection law, and the decision made by the authorities is not that which appears to us consonant with reason and justice. It is but natural that those whose proceedings it was intended to regulate by the operation of this law, should endeavor in every way to evade its provisions. Not only would they desire to escape the vigilance or overcome the integrity of the Examiner, but they would wish to obtain the implied sanction of his approval for their wares. An article which has passed the Custom House carries with it necessarily the endorsement of a competent Examiner, as one that may "properly, safely and without danger, be used for medicinal purposes." Such are the words of the law, and it is readily seen that such a sanction is worth more to a nostrum-dealer than a certificate from "the Duke of Aldborough," or a half dozen of the reverend gentlemen, who are, with us, the general sponsors of quackery.

The facts of the case are these. A quantity of certain substances labelled as Holloway's Pills and Ointment, was entered at the Custom House of this city, and referred to the drug-examiner for inspection. The duty of that officer, under the law, is to try all drugs by the standard of the United States, London, Edinburgh, French and German Pharmacopœias, and to refuse to pass all that are adulterated or deteriorated, and "improper, unsafe, or dangerous to be used for medicinal purposes." In the present instance, articles were presented of a compound character, and of the composition of which the Examiner was necessarily ignorant. They were totally unknown to the medical profession. They might contain injurious or even poisonous constituents, not discernible by the most careful examination and analysis. At all events, they were secret nostrums and not drugs or medicines recognized by any codex whatever. Had they been unofficinal simples, vegetable or mineral, the source and character of which were known, or could be readily ascertained, there could be no difficulty about them. But they were vile quackeries and impostures, compounded by a designing person to defraud a gullible public. Could any honorable and conscientious examiner give them the sanction of his endorsement? We say No!—and personally, we would have resigned the office, if the case were ours, rather than affix our name and sanction to the invoice. The Examiner, did what we think was his duty in the premises, and refused to pass the articles, referring the case thereby to his official superiors.

But his judgment has been reversed. The quackeries in question have been admitted, and now are heralded with flaming advertisements in our newspapers. Worst of all, they have have been admitted mainly on the authority of an opinion to that effect, signed by five respectable gentlemen of our city, all of whom are in official positions (we believe) in our College of Pharmacy, and one of whom is a graduate and practitioner of medicine! We give this document that there may be no mistake about it. The signatures we omit. The Italics are ours.

"We, the undersigned, are of opinion that the law in reference to adulterated drugs, medicines, and medicinal preparations was not designed to exclude secret medicines of foreign manufacture, provided they were *made by the party, and at the place specified in the label*, and that, therefore, if the Inspector believe that *such preparations are what they purport to be and not spurious*, he is bound to pass them.

"At the same time we would have it expressly understood that our private opinions are decidedly adverse to all secret medicines, whether of foreign or domestic origin, and in giving the above opinion, we do it entirely in justice to our sense of the intended meaning of the law, and not from a desire to favor the introduction of such medicines."

Now there are, to our mind, several curious features in the above document. In the first place, we do not see that any body of apothecaries, however intelligent, are the proper judges of the intention and interpretation of any law. The language of the act of Congress is explicit enough, and may be understood by any one. If there were technical difficulties, they would be better referred to those accustomed to deal with legal enactments of doubtful interpretation. But the principal peculiarity is, that this opinion comes from gentlemen who have hitherto professed a firm hostility to quackery, and are members of an institution which the medical profession has always respected and valued as a public safeguard against its encroachments. Their proviso in the concluding paragraph is all very well, but it is not worth the paper it is written upon to combat the ill effects of its predecessor. It will be seen that the position taken not only authorizes the unlimited introduction of foreign nostrums, but it actually converts the drug-examiner into a guardian of the interests of the manufacturer of the abominations. It virtually makes him say to "Doctor" Holloway:—"You may flood our land with your vile compounds. You may poison our people and extract from them untold sums of gold in return; and no imitation or counterfeit shall interfere with your profits. I stand here, by appointment of the Government of the United States, to protect and keep inviolate the sanctity of your labels and package-marks, and to condemn and forfeit all 'pills and ointment' which do not bear upon them those sacred characters!" Such is the inevitable operation of the position taken, for which we have no doubt that the foreign empiric and his agents here will be duly grateful.

We can comprehend such a position as this: that the nostrums in question are not drugs or medicines within the intent and meaning of the law, and are therefore not subject to inspection by the Drug-Examiner at all. Such is the position which we believe the department must ultimately take, if its officers determine that these nostrums shall continue to come in. It is an insult to the self-respect of any honorable pharmaceutist or physician to ask him to become the endorser as "safe and proper" of every wretched empiricism that the lust of gain may induce any unprincipled European adventurer to send over here to gull the simple Yankees with. As to the course pursued by the Examiner, we unhesitatingly pronounce it honest, manly, and consistent. That his decision has been reversed is his misfortune, *and ours.* The sting of the business is, that it has been reversed by the action of men with whom we have hitherto been happy to co-operate in the advancement of legitimate medicine and pharmacy.

